# Cytotoxic Quinones from the Roots of *Aloe dawei*

**DOI:** 10.3390/molecules19033264

**Published:** 2014-03-17

**Authors:** Negera Abdissa, Martha Induli, Paul Fitzpatrick, John Patrick Alao, Per Sunnerhagen, Göran Landberg, Abiy Yenesew, Máté Erdélyi

**Affiliations:** 1 Department of Chemistry, University of Nairobi, P.O. Box 30197, Nairobi 00100, Kenya; E-Mails: negeraabdisa@yahoo.com (N.A.); martha_induli@yahoo.com (M.I.); 2 Sahlgrenska Cancer Centre, University of Gothenburg, Gothenburg SE-405 30, Sweden; E-Mails: paul.fitzpatrick@gu.se (P.F.); goran.landberg@gu.se (G.L.); 3 Department of Chemistry and Molecular Biology, University of Gothenburg, Gothenburg SE-412 96, Sweden; E-Mails: john.p.alao@cmb.gu.se (J.P.A.); Per.Sunnerhagen@cmb.gu.se (P.S.); 4 Swedish NMR Centre, University of Gothenburg, Gothenburg SE-405 30, Sweden

**Keywords:** *Aloe dawei*, Asphodelaceae, naphthoquinone, anthraquinone, 6-hydroxy-3,5-dimethoxy-2-methyl-1,4-naphthoquinone, cytotoxicity, MCF-7

## Abstract

Seven naphthoquinones and nine anthraquinones were isolated from the roots of *Aloe dawei* by chromatographic separation. The purified metabolites were identified by NMR and MS analyses. Out of the sixteen quinones, 6-hydroxy-3,5-dimethoxy-2-methyl-1,4-naphthoquinone is a new compound. Two of the isolates, 5,8-dihydroxy-3-methoxy-2-methylnaphthalene-1,4-dione and 1-hydroxy-8-methoxy-3-methylanthraquinone showed high cytotoxic activity (IC_50_ 1.15 and 4.85 µM) on MCF-7 breast cancer cells, whereas the others showed moderate to low cytotoxic activity against MDA-MB-231 (ER Negative) and MCF-7 (ER Positive) cancer cells.

## 1. Introduction

The Alooideae subfamily of Asphodelaceae comprises seven genera with approximately 650 species [1]. Of these, the 400 species of the genus *Aloe* typically grow in temperate and subtropical parts of Africa [2,3,4]. Based on morphological characteristics [5] this genus has been divided into twenty subgroups, ranging from grass to tree *Aloe*s. While this morphology-based grouping has advantages, it does not however necessarily reflect genetic relationships. For instance, group 19 (shrubby *Aloes*) encompassing *Aloes* with prolonged stems shares similarities with group 5 (*Aloes* with striped perianth) in inflorescence and leaf characters, even though they differ with respect to caulescence and branching. Moreover, *A. dawei* of group 19 and *A. secundiflora* of group 14 have the morphological similarity of spotted perianth shapes. The uncertainty of the conventional morphology-based classification motivates an in depth investigation of the metabolic profile of *Aloe* species, and is expected to yield insight into their infrageneric relationships. In this regard, chemotaxonomic studies by Viljoen and van Wyk [6] have shown that the secondary metabolite profiles of the *Aloe* groups 5, 14, 16 and 19 are closely related.

The plant *Aloe dawei* (A. Berger) is widely distributed in Kenya, Uganda, Tanzania and Rwanda. It is named after M.T. Dawe, curator of the Botanical Gardens at Entebbe, Uganda, who described *Aloes* as plants having “leaves armed with pungent reddish-brown teeth” [7]. In Rwanda the leaf extract of *A. dawei* is indigenously used to cure malaria, whilst its leaf sap is applied in the treatment of ear inflammation [8]. Despite its use in traditional medicine, no phytochemical analysis of the plant has been carried out yet. The first analysis, including isolation, spectroscopic characterization and cytotoxic assessment of sixteen quinones, of which one is new, is reported herein.

## 2. Results and Discussion

The air dried roots of *Aloe dawei* were extracted with MeOH/CH_2_Cl_2_ (1:1) by cold percolation at room temperature. The extract was subjected to column chromatography on oxalic acid impregnated silica gel resulting in the isolation of sixteen metabolites.

**Figure 1:**
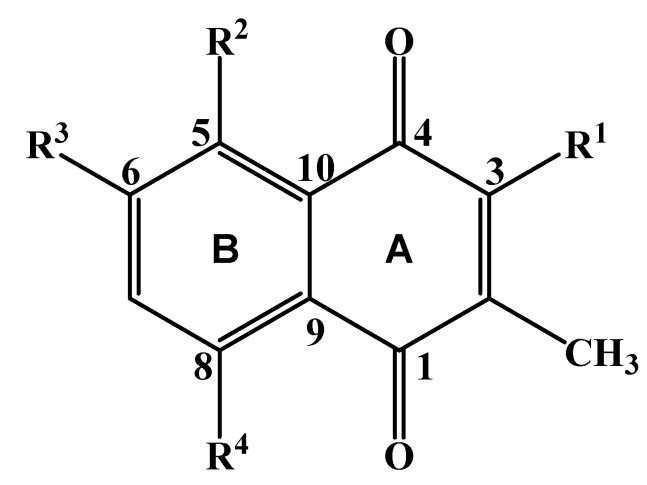
Naphthoquinones isolated from *Aloe dawei*. For 6-hydroxy-3,5-dimethoxy-2-methyl-1,4-naphthoquinone (**1**) R^1^ = R^2^ = OCH_3_, R^3^ = OH, R^4^ = H; for ancistroquinone C (**2**) R^1^ = OH, R^2^ = R^3^= OCH_3_, R^4^ = H; for 5,8-dihydroxy-3-methoxy-2-methyl-1,4-naphthoquinone (**3**) R^1^ = OCH_3_, R^2^ = R^4^= OH, R^3^ = H; for malvone A (**4**) R^1^ = OCH_3_, R^2^ = R^3^= OH, R^4^ = H; for droserone (**5**) R^1^ = R^2^ = OH, R^3^= R^4^= H; for droserone-5-methyl ether (**6**) R^1^ = OH, R^2^ = OCH_3_, R^3^= R^4^= H; for hydroxydroserone (**7**) R^1^ = R^2^ = R^4^ = OH, R^3^ = H.

Compound **1** (Figure 1) was obtained as a yellow amorphous solid. Its HR(ESI)MS analysis suggested the molecular formula C_13_H_12_O_5_ (observed *m/z* 247.0643 [M-H]^−^, expected 247.0607). Its UV absorption maxima at 225, 260, 285 and 350 nm along with the presence of two carbonyl (δ_C_ 183.0 and 187.2 ppm) and eight aromatic ^13^C-NMR signals (Table 1) indicated a 1,4-naphthoquinone skeleton [9]. The three ^1^H NMR singlets each integrating for three protons at δ_H_ 3.95, 3.77 and 1.91 ppm gave HSQC correlations to carbons at δ_C_ 63.5, 63.6, and 12.0 ppm, respectively, and correspond to two methoxy and one methyl substituents. Furthermore the ^13^C-NMR signals at δ_C_ 150.2, 160.0 and 161.4 ppm revealed three oxygenated quaternary carbons and hence confirming the presence of a dimethoxy-, methyl- and hydroxyl-substituted naphthoquinone. Two *ortho*-coupled (*J* = 8.4 Hz) aromatic protons at δ_H_ 7.19 ppm (H-7) and 7.65 ppm (H-8) suggested a 5,6-disubstituted B ring. The singlet at δ_H_ 1.91 ppm was diagnostic for a methyl substituent at the quinoid A ring that showed HMBC correlation to δ_C_ 131.5 ppm (C-2) [9,10]. A ^3^*J*_C,H_ correlation of δ_H_ 7.65 ppm (H-8) and δ_C_ 187.2 ppm allowed the assignment of the latter carbonyl to C-1. The HMBC correlation of the methyl protons at δ_H_ 1.91 ppm to C-1 is consistent with its positioning at C-2, which is in agreement with biogenetic considerations [11,12]. The high chemical shift of the methoxy carbons (δ_C_ 63.5 ppm and 63.6 ppm) is consistent with a di-*ortho*-substitution pattern for the two methoxy groups, placing them at C-3 and C-5 of ring B. Consequently, the hydroxyl group can be placed at C-6 (δ_C_ 160.0 ppm). In agreement with this, the HMBC spectrum showed correlations of the OCH_3_-3 and the CH_3_-2 protons with δ_C_ 161.4 ppm (C-3) and the OCH_3_^_^5 and the H-7 with δ_C_ 150.2 ppm (C-5). This was further confirmed by the absence of NOE correlation between H-7 (δ_H_ 7.19 ppm) and OCH_3_-5 (δ_H_ 3.77 ppm), and the lack of a signal for chelation, which rules out the placement of OH at C-6. Based on this spectroscopic evidence, the new compound was characterized as 6-hydroxy-3,5-dimethoxy-2-methyl-1,4-naphthoquinone (**1**).

**Table 1:** NMR data for 6-hydroxy-3,5-dimethoxy-2-methyl-1,4-naphthoquinone (**1**) in DMSO-*d*_6_.

	*δ*_C_	*δ*_H_ (I, *m*, *J* in Hz)	HMBC (^2^*J*, ^3^*J*)
1	187.2		
2	131.5		
3	161.4		
4	183.0		
5	150.2		
6	160.0		
7	123.6	7.19 (1H, *d*, 8.4)	C-6, C-5, C-9
8	126.5	7.65 (1H, *d*, 8.4)	C-1, C-10
9	127.4		
10	127.9		
2-CH_3_	12.0	1.91 (3H, *s*)	C-1, C-2, C-3
3-OCH_3_	63.5	3.95 (3H, *s*)	C-3
5-OCH_3_	63.6	3.77 (3H, *s*)	C-5

Six additional known naphthoquinones **2**–**7** were also isolated from the extract. They were spectroscopically identified as ancistroquinone C (**2**), previously isolated from stress-induced cell culture of *Ancistrocladus abbreviates* [9]; 5,8-dihydroxy-3-methoxy-2-methyl-1,4-naphthoquinone (**3**), recently reported from the roots of *Aloe secundiflora* [13], malvone A (**4**) [9,14], droserone (**5**) [15,16,17], droserone-5-methyl ether (**6**) [18] and hydroxydroserone (**7**) [19,20,21].

It should be emphasized that this finding is the second report on the occurrence of naphthoquinones in the genus *Aloe* [13] and the third in the family Asphodelaceae following the work of Todorova *et al.* [22].

The additional isolated compounds were identified by 2D-NMR and MS as the anthraquinones chrysophanol (**8**) [23], helminthosporin (**9**) [24], aloesaponarin I (**10**) and II (**11**) [25], laccaic acid D-methyl ester (**12**) [25,26], deoxyerythrolaccin (**13**) [27], 1-hydroxy-8-methoxy-3-methylanthraquinone (**14**) [28], and the preanthraquinones aloesaponol I (**15**) and aloesaponol II-6-methyl ether (**16**) [25,26,29]. These compounds are typical constituents of the roots of *Aloe* species and are of scarce chemotaxonomic importance at the intrageneric level [30]. On the other hand, the occurrence of naphthoquinones in *A. dawei* of *Aloe* group 19 and *A. secundiflora* of group 14 supports the previous suggestion of the close relationship between these taxa [6]. The impact of naphthoquinones in establishing the chemotaxonomic relation of *Aloe* groups should be the target of future phytochemical studies.

Quinones, in particular naphthoquinones, have raised distinct toxicological and pharmacological interest. The quinone core, presumably capable of modulating oxidative biochemical processes [31], is a common structural element in cancer chemotherapeutic agents such as doxorubicin, mitomycin C, and mitoxantrone. A number of herbal quinone metabolites have been reported to possess cytotoxic activity [32,33,34,35,36]. Compounds **1**–**16** were therefore assayed for activity against the MDA-MB-231 (ER negative) and MCF-7 (ER positive) breast cancer cell lines (Table 2). Of the isolated compounds, **3** and **14** showed strong cytotoxicity against MCF-7 cells. Compounds **4**, **11**, **13**, **15** and **16** possessed medium cytotoxicities, at least at one of the two studied cancer cell lines, whereas most constituents had low cytotoxicity.

## 3. Experimental

### 3.1. General Information

UV/Vis spectra were obtained on a Pye-Unicam SPS 150 spectrophotometer. LC-ESI-MS spectra was acquired using a Perkin Elmer PE SCIEX API 150 EX instrument equipped with a Turbolon spray ion source and a Gemini 5 mm C-18 110 Ǻ HPLC column using a water-acetonitrile gradient (80:20 to 20:80). The spectra were acquired with 30 electron volt (eV) ionization. High-resolution mass spectral analysis (Q-TOF-MS) was done by Stenhagen Analyslab AB (Gothenburg, Sweden), using a Micromass QTOFmicro instrument with lockmass-ESI source and negative ion detection. ^1^H- and ^13^C-NMR spectra were acquired for DMSO-*d*_6_ (99.8%, Sorbent AB, Gothenburg, Sweden) solutions using Varian Inova 800 MHz (^1^H: 799.87 MHz, ^13^C: 201.15 MHz) and 600 MHz (^1^H: 599.77 MHz; ^13^C: 150.83 MHz), Varian VNMR-S 500 MHz (^1^H: 499.58 MHz; ^13^C: 125.71 MHz) or Bruker 500 MHz (^1^H: 500.01 MHz, ^13^C: 125.74 MHz) spectrometers. Chemical shifts were referenced indirectly to tetramethylsilane via the residual solvent signals (DMSO, ^1^H at 2.50 ppm and ^13^C at 39.52 ppm). Spectra were processed using the software MestReNova (v 8.1.2). Full assignation was performed using ^1^H, ^13^C, COSY [38], NOESY [39], HSQC [40], HMBC [41] spectra.

**Table 2:** Cytotoxicity of the roots constituents of *Aloe dawei*.

	Isolated compound	IC_50 _(μM) ^a^	
		MCF-7 ^b^	MDA-MB-231
**1**	6-Hydroxy-3,5-dimethoxy-2-methyl-1,4-naphthoquinone	>403	>403
**2**	Ancistroquinone C	>370	330
**3**	5,8-Dihydroxy-3-methoxy-2-methylnaphthalene-1,4-dione	1.15	408
**4**	Malvone A	222	65
**5**	Droserone	>490	>490
**6**	Droserone-5-methyl ether	>459	>459
**7**	Hydroxydroserone	432	>455
**8**	Chrysophanol	>394	>394
**9**	Helminthosporin	>370	>370
**10**	Aloesaponarin I	211	>357
**11**	Aloesaponarin II	157	72
**12**	Laccaic acid D-methyl ester	>305	277
**13**	Deoxyerythrolaccin	178	140
**14**	1-Hydroxy-8-methoxy-3-methylanthraquinone	4.85	>100
**15**	Aloesaponol I	>352	125
**16**	Aloesaponol II-6-methyl ether	261	131

^a^ IC_50_: cytotoxic concentration. The mean values of at least three independent experiments are given. 95% Confidence interval is given in the Supporting Information; ^b^ As positive control 1-isopropyl-3-(pyridin-4-ylethynyl)-1H-pyrazolo[3,4-d]pyrimidin-4-amine [37] (IC_50_ = 5.0 nM, confidence interval (95%) = 1.4–17.8 nM) was used.

### 3.2. Plant Material

The roots of *Aloe dawei* were collected from Nyalenda primary school, Awendo, Kenya in February 2012. The plant was identified by Patrick Chalo Mutiso of the University of Nairobi, School of Biological Science herbarium where a voucher specimen has been deposited with deposit number NAA2012/01.

### 3.3. Extraction and Isolation

The air dried and ground roots of *Aloe dawei* (1.4 kg) were exhaustively extracted with MeOH/CH_2_Cl_2_ (1:1) by cold percolation at room temperature. The extract was evaporated under reduced pressure to yield a reddish-brown crude extract (31.0 g). The extract was subjected to column chromatography on oxalic acid impregnated silica gel (300 g) eluting with *n*-hexane containing increasing amounts of ethyl acetate to afford 34 fractions. The fraction eluted with 1% EtOAc in *n*-hexane gave 5,8-dihydroxy-3-methoxy-2-methylnaphthalene-1,4-dione (**3**, 9.2 mg) and helminthosporin (**9**, 12.3 mg). The fraction eluted with 3% EtOAc in *n*-hexane, after purification by column chromatography on Sephadex LH-20 (MeOH/CH_2_Cl_2_; 1:1), afforded hydroxydroserone (**7**, 9.4 mg) and chrysophanol (**8**, 11.8 mg). Fractions eluted with 5%–8% EtOAc in *n*-hexane contained a mixture of four compounds and were combined and purified by column chromatography on oxalic acid impregnated silica gel (increasing gradient of EtOAc in *n*-hexane) and gave droserone (**5**, 8.8 mg), 1-hydroxy-8-methoxy-3-methylanthraquinone (**14**, 32.7 mg), malvone A (**4**, 26.4 mg) and droserone-5-methyl ether (**6**, 13.5 mg). Fractions eluted with 10%–18% EtOAc in *n*-hexane gave aloesaponarin I (**10**, 14.3 mg) and aloesaponarin II (**11**, 17.8 mg) by purification of the combined fractions on Sephadex LH-20 (MeOH/CH_2_Cl_2_; 1:1). Fractions eluted with 20%–30% of EtOAc in *n*-hexane were combined and purified on Sephadex LH-20 (MeOH/CH_2_Cl_2_; 1:1) and subsequently on oxalic acid impregnated silica gel (increasing gradient of EtOAc in *n*-hexane) which gave **1** (9.8 mg), **2** (7.5 mg), deoxyerythrolaccin (**13**, 7.7 mg), aloesaponol I (**15**, 4.2 mg), laccaic acid D-methyl ester (**12**, 11.9 mg) and aloesaponol II-6-methyl ether (**16**, 15.7 mg). All compounds were obtained with >95% purity.

### 3.4. Cytotoxicity Assay

MCF-7 and MDA-MB-231, human breast cancer cells were cultured in Dulbecco’s modified eagle medium (DMEM) supplemented with 10% (*v*/*v*) fetal bovine serum, 2 mM L-glutamine, 100 units/mL penicillin and 100 μg/mL streptomycin at 37 °C in humidified 5% CO_2_. For cytotoxicity assays, cells were seeded in 96-well plates at optimal cell density (10000 cells per well) to ensure exponential growth for the duration of the assay. After a 24 h preincubation growth, the medium was replaced with experimental medium containing the appropriate drug concentrations or vehicle controls (0.1% or 1.0% *v*/*v* DMSO). After 48 h (MCF-7 cells) 72 h (MDA-MB-231 cells) incubation, cell viability was measured using Presto Blue (MCF-7 cells) or Alamar Blue reagent (MDA-MB-231 cells) (Invitrogen Ab, Lidingö, Sweden) according to the manufacturer’s instructions. Absorbance was measured at 570 nm with 600 nm as a reference wavelength. Results were expressed as the mean ± standard error for six replicates as a percentage of vehicle control (taken as 100%). Experiments were performed independently at least six times. Statistical analyses were performed using a two-tailed Student’s *t*-test. *p* < 0.05 was considered to be statistically significant

## 4. Conclusions

Phytochemical analysis of the roots of *Aloe dawei* revealed its rich anthraquinone and naphthoquinone metabolite content. Whereas anthraquinones are common chemotaxonomic markers for *Aloe* species, naphthoquinones are reported here for the second time for this genus. A new naphthoquinone, 6-hydroxy-3,5-dimethoxy-2-methyl-1,4-naphthoquinone, is reported. Two of the quinones, 5,8-dihydroxy-3-methoxy-2-methylnaphthalene-1,4-dione and 1-hydroxy-8-methoxy-3-methylanthraquinone, showed strong cytotoxicity against MCF-7 breast cancer cells. All other isolates of *A. dawei* possessed low or medium cytotoxicity. Bbosa *et al.* [42] reported 7.9 µg/mL antiplasmodial activity of the ether extract of the leaves of *A. dawei* justifying its traditional medicinal use, without evaluating its toxicity however. While the cytotoxicity of the root extract reported here does not provide any direct information on the possible toxicity of the leaves extract, nevertheless our observation highlights the need for careful evaluation of *A. dawei* extracts used in indigenous medicine to ensure patient safety.

## Supplementary Materials

Supplementary materials can be accessed at: http://www.mdpi.com/1420-3049/19/3/3264/s1.
