# Supplementary material for: Cytotoxic Quinones from the Roots of Aloe dawei
Source: Molecules. 2014 Mar 17;19(3):3264–73. doi: 10.3390/molecules19033264 (PMC6270816; doi:10.3390/molecules19033264)
Supplement: Supplementary file 1 [file molecules-19-03264-s001.pdf]

Supporting Information

1. Spectroscopic Data

6-Hydroxy-3,5-dimethoxy-2-methyl-1,4-naphthoquinone (**1**). Yellow amorphous solid. UV λ_{max} (MeoH) nm: 225, 260, 285, 350. ^1H and ^{13}C NMR see Table 1. IR ($CHCl_3$) cm^{-1}: 3605, 3020, 1504, 1418, 1211. HRESIMS *m/z* = 247.0643, $[M-H]^-$ (calculated for $C_{13}H_{12}O_5$, 247.0607).

3-Hydroxy-5,6-dimethoxy-2-methyl-1,4-naphthoquinone (**2**), *ancistroquinone C* [1]. Yellow solid. ^1H NMR (DMSO-d_6, 25 °C) δ (499.88 MHz, ppm): 1.88 (3H, *s*, CH_3-2), 3.78 (3H, *s*, OCH_3-6), 3.91 (3H, *s*, OCH_3-5), 7.43 (1H, *d*, *J* = 7.7 Hz, H-7), 7.78 (1H, *d*, *J* = 7.7 Hz, H-8); ^{13}C NMR (DMSO-d_6, 25 °C) δ (125.71 MHz, ppm): 8.4 (CH_3-2), 56.3 (OCH_3-5), 60.5 (OCH_3-6), 116.8 (CH-7), 117.9 (C-2), 122.9 (C-4a), 123.3 (CH-8), 125.6 (C-1a), 148.6 (C-6), 155.9 (C-3), 157.5 (C-5), 179.6 (C-4), 183.8 (C-1). ESI-MS (30 eV): *m/z* 271.6 $[M+Na]^+$, 249.4 $[M+H]^+$ (100), 231.2 (35), 203.5 (70).

5,8-Dihydroxy-3-methoxy-2-methyl-1,4-naphthoquinone (**3**) [2]. Red solid, ^1H NMR (DMSO-d_6, 25 °C) δ (499.88 MHz, ppm): 2.00 (3H, *s*, CH_3-2), 4.06 (3H, *s*, OCH_3-3), 7.33 (2H, *s*, H-6 & H-7), 12.02 (1H, br s, OH-5), 12.54 (1H, br s, OH-8); ^{13}C-NMR (DMSO-d_6, 25 °C) δ (125.71 MHz, ppm): 8.86 (C-2), 61.2 (OCH_3-3), 111.1 (C-1a/C-8a), 111.6 (C-8a/C-1a), 128.7 (C-6), 129.5 (C-7), 131.9 (C-2), 156.1 (C-5/C-8), 156.8 (C-8/C-5), 158.1 (C-3), 183.1 (C-4), 188.6 (C-1); ESI-MS (30 eV): *m/z* 235.0 $[M+H]^+$ (100), 207.2 (12).

2-Methyl-3-methoxy-5,6-dihydroxy-1,4-naphthoquinone (**4**), malvone A [3]. ^1H-NMR (DMSO-d_6, 25 °C) δ (499.88 MHz, ppm): 1.95 (3H, *s*, CH_3-2), 3.97 (3H, *s*, CH_3-3), 7.09 (1H, *d*, *J* = 7.1 Hz, H-7), 7.41 (1H, *d*, *J* = 7.1 Hz, H-8); ^{13}C-NMR (DMSO-d_6, 25 °C) δ (125.71 MHz, ppm): 9.4 (CH_3-2), 60.9 (OCH_3-3), 114.8 (C-10), 119.8 (C-7), 120.2 (C-8), 122.4 (C-9), 133.1 (C-2), 150.0 (C-5), 152.4 (C-6), 157.0 (C-3), 183.4 (C-4), 186.0 (C-1); ESI-MS (30 eV): *m/z* 235.0 $[M+H]^+$ (100), 207.2 (25), 189.5 (20).

2-Methyl-3,5-dihydroxy-1,4-naphtoquinone (**5**), droserone [4]. Orange solid, ^1H NMR (DMSO-d_6, 25 °C) δ (799.87 MHz, ppm): 1.92 (3H, *s*, CH_3-2), 7.25 (1H, *dd*, *J* = 1.1, 8.4 Hz, H-6), 7.49 (1H, *dd*, *J* = 1.1, 7.5 Hz, H-8), 7.69 (1H, dd, H = 7.5, 8.4 Hz, H-7), 11.38 (1H, *s*, OH-5); ^{13}C NMR (DMSO-d_6, 25 °C) δ (150.83 MHz, ppm): 8.7 (CH_3-2), 113.6 (C-10), 118.3 (C-8), 120.5 (C-2), 122.9 (C-6), 132.4 (C-9), 136.9 (C-7), 155.3 (C-3), 156.0 (C-5), 184.1 (C-1), 184.5 (C-4). ESI-MS (30 eV): *m/z* 205.1 $[M+H]^+$ (100), 149.5 (40).

2-Methyl-3-hydroxy-5-methoxy-1,4-naphtoquinone (**6**), droserone-5-methyl ether [5]. ^1H-NMR (DMSO-d_6, 25 °C) δ (799.87 MHz, ppm): 1.88 (3H, *s*, CH_3-2), 3.92 (3H, *s*, OCH_3-5), 7.46 (1H, *d*, *J* = 8.5 Hz, H-6), 7.60 (1H, *d*, *J* = 7.5 Hz, H-8), 7.75 (1H, *dd*, *J* = 7.5, 8.5 Hz, H-7), 10.64 (1H, br s, OH-3); ^{13}C NMR (DMSO-d_6, 25 °C) δ (150.83 MHz, ppm): 8.3 (CH_3-2), 56.4 (OCH_3-5), 117.0 (C-10), 117.8 (C-8), 118.2 (C-6), 134.2 (C-9), 135.8 (C-7), 155.9 (C-3), 159.4 (C-5), 178.9 (C-4), 184.5 (C-1); ESI-MS (30 eV): *m/z* 219.4 $[M+H]^+$ (100), 201.5 (28), 173.2 (50).

2-Methyl-3,5,8-trihydroxy-1,4-naphtoquinone (**7**), hydroxydroserone [6]. ^1H NMR (DMSO-d_6, 25 °C) δ (799.87 MHz, ppm): 1.88 (3H, *s*, CH_3-2), 7.28 (1H, *d*, *J=9.4 Hz*, H-6/7), 7.34 (1H, *d*, *J* = 9.4 Hz,

H-6/7), 12.90 (1H, *br s*, OH), 11.7 (1H, *br s*, OH); ^{13}C-NMR (DMSO-d$_{6,}$ 25 °C) δ (150.83 MHz, ppm): 8.1 (CH_3-2),110.8 (C-9/10), 110.9 (C-9/10), 120.4 (C-2), 127.4 (C6/7), 130.0 (C6/7), 155.4 (C-5/8), 156.0 (C-5/8), 156.8 (C-3), 182.8 (C-4), 189.0 (C-1); ESI-MS (30 eV): *m/z* 221.4 $[M+H]^+$ (35), 149.6 (40).

2-Methyl-4,5-dihydroxy-9,10-anthraquinone (**8**), chrysophanol [7]. ^{1}H NMR (DMSO-d$_{6,}$ 25 °C) δ (499.88 MHz, ppm): 2.44 (3H, *s*, CH_3-2),7.22 (1H, *d*, *J* = 1.6 Hz, H-3), 7.38 (1H, *dd*, *J* = 1.1, 8.4 Hz, H-6), 7.55 (1H, *d*, *J* = 1.6 Hz, H-1), 7.71 (1H, *dd*, *J* = 1.1, 7.5 Hz, H-8), 7.80 (1H, *dd*, *J* = 7.5, 8.4 Hz, H-76), 11.95 (1H, br s, OH); ^{13}C NMR (DMSO-d$_{6,}$ 25 °C) δ (125.71 MHz, ppm): 21.6 (CH_3-2), 113.8 (C-4a), 115.9 (C-5a), 119.2 (C-8), 120.5 (C-1), 124.1 (C-3), 124.5 (C-6), 133.0 (C-1a), 133.3 (C-8a), 137.3 (C-7), 149.1 (C-2), 161.4 (C-5), 161.7 (C-4), 181.5 (C-9), 191.5 (C-10); ESI-MS (30 eV): *m/z* 255.4 $[M+H]^+$ (33), 149.5 (100).

2-Methyl-4,5,8-trihydroxy-9,10-anthraquinone (**9**), helminthosporin [8]. ^{1}H NMR (DMSO-d$_{6,}$ 25 °C) δ (399.95 MHz, ppm): 2.45 (3H, *s*, CH_3-2), 7.26 (1H, *d*, *J* = 1.3 Hz, H-3), 7.44 (2H, *m*, H6 & H7), 7.65 (1H, *d*, *J* = 1.3 Hz, H-1), 12.1 (1H, *br s*, OH), 12.9 (1H, *br s*, OH); ^{13}C NMR ($CDCl_{3,}$ 25 °C) δ (125.71 MHz, ppm): 22.3, 112.5, 112.8, 114.0, 120.8, 124.6, 129.5, 129.6, 133.2, 149.1, 157.6, 158.2, 162.8, 186.6, 190.6. EI-MS (30 eV): *m/z* 270.0 $[M]^+$ (100).

3,8-Dihydroxy-1-methyl-9,10-anthraquinone-2-carboxylic acid methyl ester (**10**), aloesaponarin I [9]. ^{1}H NMR (DMSO-d$_{6,}$ 25 °C) δ (499.88 MHz, ppm): 2.58 (3H, *s*, CH_3-1), 3.88 (3H, *s*, OCH_3-2), 7.3 (1H, m, H-7), 7.54–7.62 (2H, m, H-6 & H-4), 7.70 (1H, m, H-5), 11.71 (1H, *br s*, OH-8), 12.78 (1H, *br s*, OH-3); ^{13}C NMR (DMSO-d$_{6,}$ 25 °C) δ (125.71 MHz, ppm): 19.9 (C-1), 52.5 (OCH_3-2), 112.0 (C-4), 116.7 (C-8a), 118.3 (C-6), 122.5 (C-2), 124.4 (C-7), 129.6 (C-1a), 132.3 (C-5a), 136.1 (C-5), 136.7 (C-1), 141.0 (C-1), 158.9 (C-3), 161.4 (C-8), 167.2 ($\underline{C}OOCH_3$), 181.8 (C-10), 189.2 (C-9); ESI-MS (30 eV): *m/z* 313.2 $[M+H]^+$ (100), 281.4 (89).

3,8-Dihydroxy-1-methyl-9,10-anthraquinone (**11**), aloesaponarin II [9]. ^{1}H NMR (DMSO-d$_{6,}$ 25 °C) δ (499.88 MHz, ppm): 2.70 (3H, *s*, CH_3-1), 7.04 (1H, *d*, *J* = 2.5 Hz, H-2), 7.31 (1H, *dd*, *J* = 1.2, 8.5 Hz, H-7), 7.44 (1H, *d*, *J* = 2.5 Hz, H-4), 7.62 (1H, *dd*, *J* = 1.2, 7.5 Hz, H-5), 7.71 (1H, *dd*, *J* = 7.5,8.5 Hz, H-6), 11.1 (1H, *br s*, OH), 13.0 (1H, *s*, OH); ^{13}C NMR (DMSO-d$_{6,}$ 25 °C) δ (125.71 MHz, ppm): 23.6 (C-1), 112.1 (C-4), 116.5 (C8a), 118.3 (C-5), 122.5 (C-1a), 124.3 (C-7), 124.6 (C-2), 132.6 (C-5a), 136.1 (C-6), 137.0 (C-4a), 145.5 (C1), 161.2 (C-8), 162.4 (C-3), 182.3 (C-10), 189.4 (C-9); ESI-MS (30 eV): *m/z* 255.6 $[M+H]^+$ (100), 149.5 (60).

3,6,8-Trihydroxy-1-methyl-9,10-anthraquinone-2-carboxylic acid methyl ester (**12**), laccaic acid-D-methyl ester [10,11]. ^{1}H NMR (DMSO-d$_{6,}$ 25 °C) δ (499.88 MHz, ppm): 2.54 (3H, s, CH_3-1), 3.84 (3H, s, OCH_3-2), 6.53 (1H, d, *J* = 2.4 Hz, H-5), 6.99 (1H, *d*, *J* = 2.4 Hz, H-7), 7.53 (1H, s, H-4), 11.3 (1H, *br s* , OH), 13.0 (1H, *s*, OH-8); ^{13}C NMR (DMSO-d$_{6,}$ 25 °C) δ (125.71 MHz, ppm): 20.6 (C-1), 52.8 (OCH_3-2), 107.6 (C-7), 108.7 (C-5), 110.4 (C-8a), 112.3 (C-4), 122.8 (C-1a), 130.0 (C-4a), 134.3 (C-5a), 141.0 (C-1), 158.5 (C-3), 164.4 (C-6), 164.8 (C-8), 167.7 (COO-2), 182.1 (C-10), 188.0 (C-9); ESI-MS (30 eV): *m/z* 329.5 $[M+H]^+$ (100), 297.7 (70).

3,6,8-Trihydroxy-1-methyl-9,10-anthraquinone (**13**), deoxyerythrolaccin [12]. ^{1}H NMR ($DMSO-d_6$, 25 °C) δ (499.88 MHz, ppm): 2.70 (3H, s, CH_3-1), 6.56 (1H, *d*, *J* = 2.4 Hz, H-7), 7.03 (1H, *d*, *J* = 2.6 Hz, H-2), 7.43 (1H, *d*, *J* = 2.4 Hz, H-5), 7.40 (1H, *d*, *J* = 2.6 Hz, H-3, 11.01 (1H, *br s*, OH), 13.24 (1H, s, OH-8); ^{13}C NMR ($DMSO-d_6$, 25 °C) δ (125.71 MHz, ppm): 23.6 (C-1), 107.1 (C-5), 108.2 (C-7), 110.0 (C-8a), 112.1 (C-4), 122.4 (C-1a), 124.6 (C-2), 134.3 (C-5a), 136.7 (C-4a), 145.0 (C1), 161.8 (C-3), 164.1 (C-6), 164.5 (C-8), 182.4 (C-10), 188.0 (C-9). ESI-MS (30 eV): *m/z* 271.6 $[M+H]^+$ (100), 149.5 (8).

6-Hydroxy-8-methoxy-3-methyl-1,8-anthraquinone (**14**) [13]. ^{1}H NMR ($DMSO-d_6$, 25 °C) δ (799.87 MHz, ppm): 2.41 (3H, s, CH_3-2), 3.97 (3H, s, OCH_3-5), 7.16 (1H, *s*, H-3), 7.46 (1H, *s*, H-1), 7.61 (1H,*d*, *J* = 8.4 Hz, H-8), 7.79 (1H, *d*, *J* = 7.6 Hz, H-6), 7.86 (1H, *dd*, *J* = 7.6, 8.4 Hz, H-7), 12.9 (1H, *br s*, OH-1); ^{13}C NMR ($DMSO-d_6$, 25 °C) δ (125.71 MHz, ppm): 21.5 (CH_3-2), 56.6 (OCH_3-5), 114.6 (C-4a), 119.3 (C-6), 119.4 (C-8), 119.8 (C-5a), 124.1 (C-3), 132.1 (C-1a), 134.9 (C-8a), 136.2 (C-7), 147.5 (C-2), 160.6 (C-5), 161.6 (C-7), 182.3 (C-10), 187.7 (C-9); ESI-MS (30 eV): *m/z* 269.3 $[M+H]^+$ (100).

3,6,9-Trihydroxy-1-methyl-8-oxo-7,8-dihydro-anthracene-2-carboxylix acid methyl ester (**15**), aloespanol I [10]. ^{1}H NMR ($DMSO-d_6$, 25 °C) δ (799.87 MHz, ppm): 2.65 (1H, *dd*, *J* = 6.9, 15.7 Hz, CH_{2a}-7), 2.66 (3H, *s*, CH_3-1), 2.86 (1H, *dd*, *J* = 6.9, 15.7 Hz, CH_{2b}-7), 2.91 (1H, *dd*, *J* = 3.7, 16.9 Hz, CH_{2b}-7), 3.09 (1H, *dd*, *J* = 3.5, 16.9 Hz, CH_{2b}-7), 3.80 (3H, s, OCH_3-2), 4.21 (*dddd*, *J* = 3.5, 3.7, 6.9, 6.9 Hz, H-7), 5.17 (1H, *br s*, OH), 6.89 (1H, *s*, H-4), 6.92 (1H, *s*, H-5); ^{13}C NMR ($DMSO-d_6$, 25 °C); δ (201.14 MHz, ppm): 37.3 (C-5) 46.4 (C-7), 51.9 (OCH_3-2), 64.06 (C-6), 107.3 (C-4), 110.5 (C-8a), 115.0 (C-2), 116.4 (C-10), 126.1 (C-1a), 137.1 (C-4a), 137.2 (C-5a), 141.0 (C-2), 155.5 (C-9), 166.4 (C-3), 168.1 (COO-2), 203.9 (C-8); ESI-MS (30 eV): *m/z* 317.2 $[M+H]^+$ (100), 285.4 (31).

3,9-Dihydroxy-6-methoxy-8-methyl-3,4-dihydro-2H-anthracen-1-one (**16**), aloespanol II [10]. ^{1}H NMR ($DMSO-d_6$, 25 °C) δ (499.88 MHz, ppm): 1.98 (1H, *m*, H-X), 2.17 (1H, *m*, H-X), 2.43 (3H, s, CH_3-8), 2.71–2.84 (2H, *m*, H-X), 3.25 (3H, *s*, OCH_3-6), 4.77 (1H, *m*, H-3), 5.57 (1H, *d*, *J* = 5.5 Hz,OH-3), 6.81 (1H, *s*, H-5), 7.17 (1H, *s*, H-7), 7.21 (1H, *s*, H-10); ^{13}C NMR ($DMSO-d_6$, 25 °C); δ (125.71 MHz, ppm): 21.8 (CH_3-8), 31.1 (C-2), 34.9 (C-4), 55.8 (OCH_3-6), 66.3 (C-3), 108.2 (C-5), 109.3, 112.5 (C-8a), 114.6 (C-10), 119.4 (C-7), 139.9, 141.9 (C-5a), 142.1 (C-8), 159.2 (C-6), 164.9 (C-9), 204.2 (C-1); ESI-MS (30 eV): *m/z* 273.3 $[M+H]^+$ (00), 255.6 (92).

2. 95% Confidence Intervals for the Cytotoxicity Measurements

Table S1. The 95% confidence interval for the CC_{50} measurements, MCF-7 cell line.

Compound	EC_{50} (ug/mL)	95% CI (ug/mL)
1	>100	-
2	>100	-
3	0.27	0.15–0.47
4	52	17–60
5	>100	-
6	>100	-
7	95	49–185
8	>100	-
9	>100	-
10	59	35–99
11	40	27.3–59.2
12	>100	-
13	48	15–155
14	1.3	0.3–4.8
15	>100	-
16	71	-

Table S2. The 95% confidence interval for the CC_{50} measurements, MDA-MB-231 cell line.

Compound	EC_{50} (ug/mL)	95% CI (ug/mL)
1	>100	-
2	89.03	71.21–92.16
3	95.83	93.97–105.2
4	15.28	2.46–18.76
5	>100	-
6	>100	-
7	>100	-
8	>100	-
9	>100	-
10	>100	-
11	18.25	13.42–27.90
12	90.9	83.90–100. 6
13	37.82	33.62–57.67
14	>100	-
15	35.41	27.24–42.48
16	35.54	29.93–48.97

References

1. Bringmann, G.; Rudenauer, S.; Irmer, A.; Bruhn, T.; Brun, R.; Heimberger, T.; Stuhmer, T.; Bargou, R.; Chatterjee, M. Antitumoral and antileishmanial dioncoquinones and ancistroquinones from cell cultures of Triphyophyllum peltatum (Dioncophyllaceae) and Ancistrocladus abbreviatus (Ancistrocladaceae). *Phytochemistry* **2008**, *69*, 2501–2509.
2. Induli, M.; Cheloti, M.; Wasuna, A.; Wekesa, I.; Wanjohi, J.M.; Byamukama, R.; Heydenrich, M.; Makayoto, M.; Yenesew, A. Naphthoquinones from the roots of Aloe secundiflora. *Phytochem. Lett.* **2012**, *5*, 506–509.
3. Veshkurova, O.; Golubenko, Z.; Pshenichnov, E.; Arzanova, I.; Uzbekov, V.; Sultanova, E.; Salikhov, S.; Williams, H.J.; Reibenspies, J.H.; Puckhaber, L.S.; *et al*. Malvone A, a phytoalexin found in Malva sylvestris (family Malvaceae). *Phytochemistry* **2006**, *67*, 2376–2379.
4. Kreher, B.; Neszmelyi, A.; Wagner, H. Naphthoquinones from Dionaea-Muscipula. *Phytochemistry* **1990**, *29*, 605–606.
5. Ghera, E.; Bendavid, Y. Annulation Reactions Leading to Naphthalene Derivatives - New Syntheses of Natural 1,2-Naphthoquinones and 1,4-Naphthoquinones. *J Org Chem* **1985**, *50*, 3355–3359.
6. Budzianowski, J. Naphthoquinone glucosides of Drosera gigantea from in vitro cultures. *Planta Med.* **2000**, *66*, 667–669.
7. Danielsen, K.; Aksnes, D.W. Nmr-Study of Some Anthraquinones from Rhubarb. *Magn. Reson. Chem.* **1992**, *30*, 359–360.
8. Yagi, A.; Makino, K.; Nishioka, I. Studies on Constituents of Aloe-Saponaria Haw .2. Structures of Tetrahydroanthracene Derivatives, Aloesaponol-Iii and Aloesaponol-Iv. *Chem. Pharm. Bull.* **1977**, *25*, 1764–1770.
9. Makino, K.; Yagi, A.; Nishioka, I. Studies on Constituents of Aloe-Arborescens Mill Var Natalensis Berger .2. Structures of 2 New Aloesin Esters. *Chem. Pharm. Bull.* **1974**, *22*, 1565–1570.
10. Yagi, A.; Makino, K.; Nishioka, I. Studies on Constituents of Aloe-Sapnaria Haw .1. Structures of Tetrahydroanthracene Derivatives and Related Anthraquinones. *Chem. Pharm. Bull.* **1974**, *22*, 1159–1166.
11. Dagne, E.; Casser, I.; Steglich, W. Aloechrysone, a Dihydroanthracenone from Aloe-Berhana. *Phytochemistry* **1992**, *31*, 1791–1793.
12. Mehandal. A.R.; Rao, A.V.; Shaikh, I.N.; Venkatar, K. Desoxyerythrolaccin and Laccaic Acid D. *Tetrahedron. Lett.* **1968**, 2231.
13. Zaman, K.; Khan, M.R.; Ali, M.; Maitland, D.J. New anthraquinone dimer from the root bark of Cassia artemisioides (Gaudich. Ex. DC) Randell. *J. Asian Nat. Prod. Res.* **2011**, *13*, 62–67.

© 2014 by the authors; licensee MDPI, Basel, Switzerland. This article is an open access article distributed under the terms and conditions of the Creative Commons Attribution license (http://creativecommons.org/licenses/by/3.0/).
